# Glucose Plus Fructose Ingestion for Post-Exercise Recovery—Greater than the Sum of Its Parts?

**DOI:** 10.3390/nu9040344

**Published:** 2017-03-30

**Authors:** Javier T. Gonzalez, Cas J. Fuchs, James A. Betts, Luc J. C. van Loon

**Affiliations:** 1 Department for Health, University of Bath, Bath BA2 7AY, UK; j.betts@bath.ac.uk; 2 Department of Human Biology and Movement Sciences, NUTRIM School of Nutrition and Translational Research in Metabolism, Maastricht University Medical Centre+ (MUMC+), P.O. Box 616, 6200 MD Maastricht, The Netherlands; cas.fuchs@maastrichtuniversity.nl (C.J.F.); l.vanloon@maastrichtuniversity.nl (L.J.C.v.L.)

**Keywords:** carbohydrates, glycogen, liver, metabolism, muscle, resynthesis, sports nutrition, sucrose

## Abstract

Carbohydrate availability in the form of muscle and liver glycogen is an important determinant of performance during prolonged bouts of moderate- to high-intensity exercise. Therefore, when effective endurance performance is an objective on multiple occasions within a 24-h period, the restoration of endogenous glycogen stores is the principal factor determining recovery. This review considers the role of glucose–fructose co-ingestion on liver and muscle glycogen repletion following prolonged exercise. Glucose and fructose are primarily absorbed by different intestinal transport proteins; by combining the ingestion of glucose with fructose, both transport pathways are utilised, which increases the total capacity for carbohydrate absorption. Moreover, the addition of glucose to fructose ingestion facilitates intestinal fructose absorption via a currently unidentified mechanism. The co-ingestion of glucose and fructose therefore provides faster rates of carbohydrate absorption than the sum of glucose and fructose absorption rates alone. Similar metabolic effects can be achieved via the ingestion of sucrose (a disaccharide of glucose and fructose) because intestinal absorption is unlikely to be limited by sucrose hydrolysis. Carbohydrate ingestion at a rate of ≥1.2 g carbohydrate per kg body mass per hour appears to maximise post-exercise muscle glycogen repletion rates. Providing these carbohydrates in the form of glucose–fructose (sucrose) mixtures does not further enhance muscle glycogen repletion rates over glucose (polymer) ingestion alone. In contrast, liver glycogen repletion rates are approximately doubled with ingestion of glucose–fructose (sucrose) mixtures over isocaloric ingestion of glucose (polymers) alone. Furthermore, glucose plus fructose (sucrose) ingestion alleviates gastrointestinal distress when the ingestion rate approaches or exceeds the capacity for intestinal glucose absorption (~1.2 g/min). Accordingly, when rapid recovery of endogenous glycogen stores is a priority, ingesting glucose–fructose mixtures (or sucrose) at a rate of ≥1.2 g·kg body mass^−1^·h^−1^ can enhance glycogen repletion rates whilst also minimising gastrointestinal distress.

## 1. Introduction

Carbohydrates are a major substrate for oxidation during almost all exercise intensities [1]. The main determinants of carbohydrate utilisation during exercise are the intensity and duration of exercise [1,2], followed by training and nutritional status [3,4]. In the fasted state, the main forms of carbohydrate utilised during exercise are skeletal muscle glycogen and plasma glucose (derived primarily from liver glycogen and gluconeogenesis) [1]. Compared to fat stores, the capacity for humans to store carbohydrates is limited; >100,000 kcal stored as fat versus <3000 kcal stored as carbohydrate in a typical 75-kg person with 15% body fat [5]. Therefore, glycogen stores can be almost entirely depleted within 45–90 min of moderate- to high-intensity exercise [6,7], with the occurrence of fatigue strongly associated with the depletion of endogenous carbohydrate stores [8,9,10]. Nutritional strategies to complement or replace endogenous carbohydrate stores as a fuel during exercise have been studied for decades [9,11]. It is now well established that carbohydrate ingestion during exercise improves endurance performance and delays fatigue in events requiring a sustained moderate to high intensity for more than 45 min [12]. Due to the strong relationship between replenishment of liver and skeletal muscle glycogen stores with subsequent exercise tolerance [7,10], the main factor determining recovery time is the rate of glycogen repletion. This is especially relevant when optimal performance is required on more than one occasion with a limited interval between bouts, such as during intensive training periods, stage races (e.g., Tour de France) and tournament-style competitions. In the hours following exercise, carbohydrate ingestion is a requirement for substantial replenishment of skeletal muscle glycogen stores [13], and the appropriate dose of carbohydrate (or co-ingestion of protein with suboptimal carbohydrate intake) can accelerate the replenishment of skeletal muscle glycogen contents [14,15].

In recent years, there has been an increasing appreciation of the different types of carbohydrates that can be ingested during and after exercise. When large amounts of carbohydrate are ingested (>1.4 g·min^−1^) the combined ingestion of glucose and fructose can improve performance by ~1–9% over the ingestion of glucose (polymers) alone [16]. The performance benefits of glucose–fructose co-ingestion are likely due to more rapid digestion and absorption of the carbohydrate, providing exogenous fuel at a faster rate than glucose ingestion alone. Faster digestion and absorption rates of carbohydrates during recovery from exercise may also have benefits for more rapid recovery of glycogen stores post-exercise [15,17]. With this in mind, this review provides an overview of dietary carbohydrates, glycogen stores and exercise capacity, before focussing on the role of glucose–fructose mixtures in post-exercise recovery of skeletal muscle and liver glycogen stores.

## 2. Dietary Carbohydrates for Sport Nutrition

Dietary carbohydrates come in many forms, comprising monosaccharides such as glucose, fructose and galactose; disaccharides such as maltose, sucrose and lactose; and polysaccharides such as maltodextrin and starch (Table 1). The rates of digestion, intestinal absorption and hepatic metabolism of carbohydrates are key determinants of carbohydrate delivery to skeletal muscle tissue. These factors are therefore important considerations when choosing a nutritional strategy to optimize carbohydrate delivery during and after exercise.

Glucose is a constituent of most disaccharides and polysaccharides and is therefore the most ubiquitous carbohydrate in most people’s diets (Table 1). Glucose is also the primary cellular fuel source in almost all human tissues. Carbohydrates must first be hydrolysed into their constituent monomers before being absorbed across the intestine and entering the systemic circulation [23]. Therefore, most dietary carbohydrates are broken down into glucose, fructose and/or galactose prior to their subsequent absorption. The major intestinal absorption route of glucose involves sodium-dependent glucose transporter 1 (SGLT1), which transports glucose from the intestinal lumen into the enterocyte [23]. Other putative routes include transport by glucose transporter 2 (GLUT2) and GLUT12, although these are yet to be clearly established in humans [24], and are likely to play only minor roles in intestinal glucose absorption [23]. Whilst fructose has an identical chemical formula to glucose (C_6_H_12_O_6_), glucose has an aldehyde group at position 1 of its carbon chain, whereas fructose possesses a keto group in position two of its carbon chain [25]. A notable difference in the handling of fructose compared to most other carbohydrates is the primary intestinal transport protein responsible for transporting fructose from the intestinal lumen to within the enterocyte: GLUT5 (Table 1). Other fructose transporters may also be involved in fructose absorption, but again are likely to play minor roles in comparison to GLUT5 [22]. 

When ingested alone, the hydrolysis of most carbohydrates is rapid and does not limit the rate of digestion and absorption. Therefore, the rate at which glucose polymers such as maltose, maltodextrin and starch can be digested, absorbed and used as a fuel source is not substantially slower than that of glucose [26,27,28]. Furthermore, the hydrolysis of sucrose (by sucrase) is also rapid and exceeds the rate of intestinal absorption of glucose and fructose [29]. An exception to this rule is isomaltulose. Due to the different bond linking glucose and fructose, the hydrolysis rate of isomaltulose (by isomaltase) is drastically slower than that of sucrose [20,30]. Isomaltulose thereby produces a lower glycaemic and insulinaemic response following ingestion, and suppresses fat oxidation to a lesser extent than sucrose [31]. However, presumably due to this slow rate of digestion and absorption, isomaltulose exacerbates gastrointestinal distress when consumed in large amounts during exercise [32].

After intestinal absorption, the metabolism of various dietary carbohydrates also differs. In contrast to glucose, which can bypass the liver and enter the systemic circulation, fructose and galactose are almost completely metabolised upon first pass of the liver [25,33]. This splanchnic sequestration appears to be enhanced by the co-ingestion of glucose [33]. Fructose and galactose are converted in the liver into glucose, lactate, glycogen and lipids, which subsequently appear in the circulation [25,33]. The energy cost of converting fructose into glucose and other substrates is likely to account for the greater postprandial thermogenesis seen with fructose versus glucose ingestion [34]. Because of this hepatic metabolism, the blood glucose and insulin responses to fructose or galactose ingestion are attenuated when compared to glucose ingestion [35,36]. This lower insulin response may have implications for glycogen storage in recovery from exercise.

Hepatic fructose metabolism also differs from hepatic glucose metabolism in its regulation by insulin. Both glucose and fructose enter the liver via the insulin-independent transporter, GLUT2. However, hepatic glucose metabolism is then regulated by insulin and the cellular energy status [5,25]. Insulin, ATP and citrate concentrations regulate glucose flux to pyruvate via modulating the activity of hexokinase IV and glycolytic enzymes [37]. Hepatic fructose metabolism on the other hand, is independent of insulin and does not display negative feedback inhibition by ATP nor citrate [25].

## 3. Endogenous Carbohydrate Stores and Exercise Performance

### 3.1. Muscle Glycogen

The reintroduction of the muscle biopsy technique to exercise physiology in the 1960s clearly demonstrated the heavy reliance on skeletal muscle glycogen as a fuel source during exercise [8,38]. There is a strong relationship between baseline skeletal muscle glycogen contents and subsequent endurance exercise capacity [8]. Furthermore, the capacity for exercise is severely compromised when skeletal muscle glycogen stores are depleted, even when other substrate sources are available in abundance [9]. The defined mechanisms that link skeletal muscle glycogen contents and exercise tolerance are incompletely understood. It is thought that skeletal muscle glycogen is more than just a fuel source, and that glycogen also acts as a signalling molecule to control skeletal muscle cell function and regulate exercise capacity [39].

Skeletal muscle glycogen provides a rapid and efficient (energy yield per unit oxygen) fuel source for energy expenditure, such that when skeletal muscle glycogen stores are depleted, the rate of energy production is severely compromised. Clear support for the important role of glycogen as a substrate in supporting energy requirements to allow intense exercise is provided by observations of individuals with McArdle’s disease (glycogen storage disease type V; GSD5). These individuals display high skeletal muscle glycogen concentrations but an inability to utilise this glycogen as a substrate source [40], and subsequently can also display extreme intolerance to intense exercise [41]. This is partly due to glycogen oxidation resulting in maximal ATP re-synthesis rates that are >2-fold greater than fat or plasma glucose oxidation [42,43]. Therefore, when high rates of ATP re-synthesis are required over a prolonged duration, it would appear there is no substitute for glycogen as a fuel. Furthermore, the oxidation of carbohydrates is more oxygen efficient than that of fat, deriving more energy per litre of oxygen consumed [44]. Consequently, oxidising carbohydrates over fats provides an advantage in sports where the rate of oxygen delivery to active muscle is limiting to performance.

A reduced ability of glycogen to fuel metabolism may not fully account for the exercise intolerance with low skeletal muscle glycogen content. Low glycogen contents are still associated with impaired skeletal muscle function, even when ATP concentrations would be normalised [45]. Therefore, it has recently been proposed that glycogen is also an important signalling molecule that regulates sarcoplasmic reticulum calcium release rates and thus skeletal muscle function [39]. Accordingly, adequate skeletal muscle glycogen availability appears to be critically important (via multiple mechanisms) in maintaining optimal performance during prolonged bouts of moderate- to high-intensity exercise.

### 3.2. Liver Glycogen

Liver glycogen plays a central role in blood glucose homeostasis during conditions such as exercise, fasting and feeding [5]. After an overnight fast (e.g., 12 h), ~50% of plasma glucose appearance at rest is accounted for by liver glycogen utilisation, with the remainder provided by gluconeogenesis [46]. Even resting metabolic requirements can therefore deplete liver glycogen stores almost entirely within 48 h of carbohydrate restriction [47].

Plasma glucose is constantly utilised as a fuel source at rest and during almost all exercise intensities [1]. During exercise in a fasted state, plasma glucose that is taken up by skeletal muscle is continuously replaced by gluconeogenesis and glycogen degradation, predominantly derived from the liver [48]. In the absence of carbohydrate ingestion liver glycogen stores can be rapidly depleted (by ~40%–60%) within 90 min of moderate- to high-intensity (~70% VO_2_ peak) exercise [6,7,49]. The rate of liver glycogen depletion during exercise in a fasted state will depend primarily on the intensity of exercise and the training status of the individual; higher exercise intensities are associated with higher rates of liver glycogen utilisation, particularly in untrained individuals [5]. Endurance-trained athletes do not appear to store more liver glycogen than untrained individuals but endurance-type exercise training is associated with a lower rate of liver glycogen utilisation during exercise (at the same absolute or relative intensity) [5]. Therefore, endurance athletes can exercise at a given exercise intensity for longer before liver glycogen contents will reach a critically low level [5].

Few studies have directly measured the relationship between liver glycogen contents and exercise tolerance in humans. One of the only studies to have performed concomitant measures of liver glycogen content and exercise capacity demonstrated a modest positive relationship between liver glycogen repletion after an initial bout of exercise, and subsequent endurance capacity [7]. Furthermore, in this study the correlation between muscle glycogen repletion and subsequent endurance capacity was weaker than that with liver glycogen repletion, and the addition of muscle glycogen repletion to liver glycogen repletion did not further improve the relationship between liver glycogen repletion and exercise capacity [7]. Consequently, post-exercise recovery of liver glycogen stores may be at least as important as muscle glycogen stores for subsequent endurance capacity. The mechanisms by which liver glycogen contents regulate exercise capacity currently remain unknown, but given the fundamental role of hepatic metabolism in glucose homeostasis, low liver glycogen stores are likely to inhibit exercise capacity (at least in part) via a reduction in blood glucose availability and premature hypoglycaemia [5]. Liver glycogen may also act as a biological signal to regulate metabolism (and potentially exercise capacity). Rodent data suggest that liver glycogen contents modulate fatty acid availability via a liver–brain–adipose tissue axis [50]. Therefore, brain sensing of liver glycogen contents could regulate metabolism (and theoretically fatigue) during exercise.

It has been suggested that it may take longer to recover liver, compared to muscle glycogen stores post-exercise, in humans [5], which is likely due to changes in splanchnic handling of glucose in the post-exercise period. Splanchnic glucose output of an oral glucose load is ~30% at rest, but can double to ~60% post-exercise [51]. This may be partly due to greater post-exercise increases in blood flow to muscle [52], compared to the liver, resulting in relatively more ingested glucose made available to the muscle. On this basis, nutritional strategies to optimise short-term recovery from prolonged exercise should focus on both liver and muscle glycogen repletion, since both display limitations in their capacity to replenish carbohydrate stores and either could be instrumental to optimizing subsequent performance.

## 4. Physiological Rationale for Glucose–Fructose Co-Ingestion in Post-Exercise Recovery

Alongside insulin concentrations, carbohydrate delivery to the liver and skeletal muscle can be a rate limiting step in post-exercise glycogen re-synthesis, as demonstrated by >2-fold higher glycogen repletion rates with glucose infusion [53,54] compared to the highest rates ever reported with oral carbohydrate ingestion [55]. During exercise, exogenous carbohydrate oxidation can differ depending on the type of carbohydrates ingested [56]. These differences may be attributable to differences in carbohydrate digestion and absorption kinetics during exercise [56,57]. It could be hypothesised that these differences are also evident during post-exercise recovery, implying that rapidly digested and absorbed carbohydrates may accelerate recovery of endogenous glycogen stores. 

**Figure 1:**
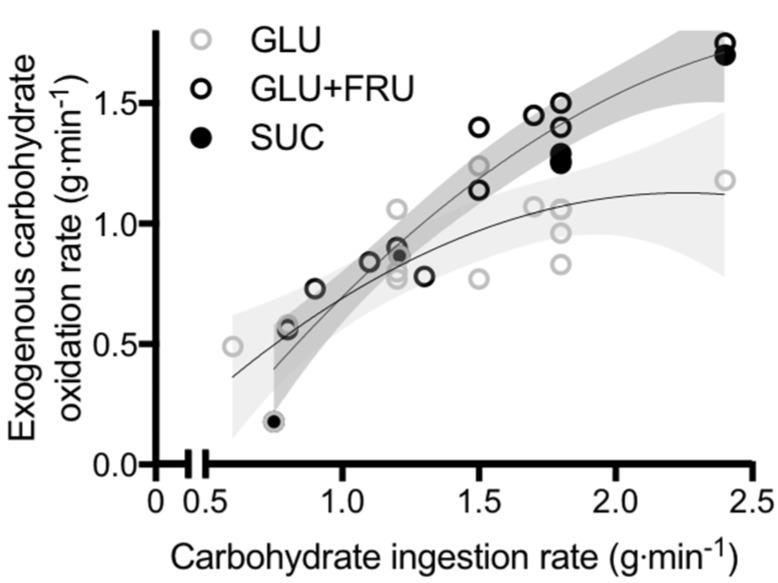
Peak exogenous carbohydrate oxidation rates during exercise in studies that directly compared glucose (polymer) ingestion alone (GLU), vs. either glucose plus fructose co-ingestion (GLU + FRU), or sucrose ingestion (SUC). Each symbol represents the mean from a single study. The light grey shaded area represents the 95% confidence intervals for GLU and the dark grey shaded area represents the 95% confidence intervals for GLU + FRU and SUC. Data extracted from references [22,23,55,56,57,58,59,60,61,62,63,64,65,66].

**Figure 2:**
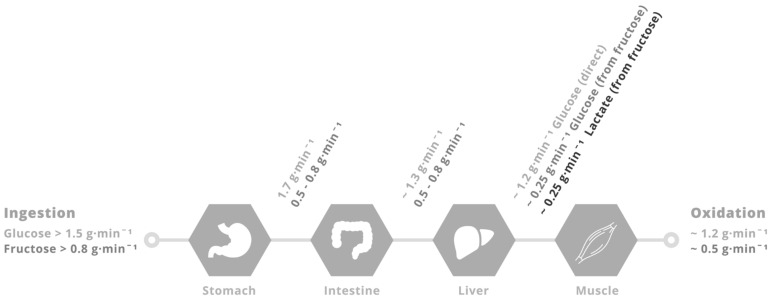
Putative limitations in carbohydrate delivery to skeletal muscle during exercise with glucose–fructose (or sucrose) co-ingestion. When large amounts of glucose (>1.5 g·min^−1^) and fructose (>0.8 g·min^−1^) are ingested during prolonged, moderate- to high-intensity (50%–70% VO_2_ peak) exercise, the rate of gastric emptying is unlikely to be limiting, since gastric emptying rates of glucose are in the region of 1.7 g·min^−1^ [67]. Rates of intestinal glucose absorption are ~1.3 g·min^−1^ [68]. Rates of glucose appearance into the peripheral circulation and subsequently oxidised are ~1.2 g·min^−1^ [58,70]. Rates of fructose (and sucrose) gastric emptying and intestinal absorption must be at least 0.5 g·min^−1^ since the appearance rate into the peripheral circulation of fructose derived carbohydrate is ~0.5 g·min^−1^ [71], with ~50% in the form of glucose and 50% in the form of lactate, that are subsequently oxidised by skeletal muscle at a rate of ~0.5 g·min^−1^ [71].

To obtain insight into the role of glucose–fructose co-ingestion on carbohydrate digestion, absorption and utilisation kinetics during exercise, we performed a literature search (PubMed, February 2017). This included the search terms “exogenous”, “carbohydrate”, “glucose”, “fructose”, “sucrose” and “oxidation”. This search was complemented by a manual search of references within papers. In order to minimize the potential for inter-subject and inter-laboratory variability, studies were limited to peer-reviewed published articles to date that have directly compared glucose (polymer) ingestion alone with glucose–fructose (sucrose) co-ingestion and determined exogenous carbohydrate oxidation rates during exercise. When ingesting glucose(polymers) during exercise, the maximal rate of exogenous carbohydrate oxidation increases in a curvilinear fashion with carbohydrate ingestion rate, reaching a peak exogenous oxidation rate of ~1.2 g·min^−1^ (Figure 1) [26,27,58,59,60,61,62,63,64,65,66,67,68,69]. The primary limitation in the rate of exogenous carbohydrate oxidation is thought to be intestinal absorption, since gastric emptying rates of glucose during exercise have been reported to exceed 1.5 g·min^−1^ [70], and when the intestine and liver are bypassed with intravenous glucose infusion, exogenous oxidation rates of 2 g·min^−1^ can be achieved [57]. Furthermore, maximal intestinal glucose absorption rates at rest have been estimated to be ~1.3 g·min^−1^ [71]. Exercise up to an intensity of 70% VO_2_ peak does not alter the intestinal absorption of glucose [72]. Therefore, it is reasonable to assume that this ~1.3 g·min^−1^ limit also applies during most exercise intensities, suggesting that intestinal absorption rather than liver glucose metabolism is the primary limitation to exogenous glucose oxidation during exercise (Figure 2) [73]. Nevertheless, this remains speculative in the absence of direct measures of intestinal absorption.

When fructose is co-ingested with glucose during exercise, exogenous carbohydrate oxidation rates of ~1.7 g·min^−1^ can be achieved; substantially higher than that seen with glucose ingestion alone (Figure 1) [26,27,58,59,60,61,62,63,64,65,66,67,68,69]. Whether glucose and fructose are ingested as sucrose or as free monosaccharides does not appear to influence the rate of exogenous carbohydrate oxidation (Figure 1). This is consistent with observations that the rate of digestion and intestinal absorption of glucose and fructose does not differ when ingested as sucrose or as co-ingestion of free glucose and free fructose [29]. Therefore, the hydrolysis of sucrose does not appear to be rate limiting to absorption of its monosaccharide products and can be used as an alternative to free glucose and free fructose. In a systematic assessment of optimal fructose:glucose ratios using dual-isotope labelling, it is apparent that a fructose:glucose ratio of 0.8-1.0-1.0 (0.67 g·min^−1^ fructose plus 0.83 g·min^−1^ glucose (polymers)) provides the greatest exogenous carbohydrate oxidation efficiency and endurance performance [74].

Fructose metabolism differs markedly from glucose metabolism. Firstly, fructose is primarily absorbed across the apical membrane of the intestinal enterocytes by different transport proteins (GLUT5, as opposed to SGLT1). Secondly, plasma fructose concentrations remain relatively low (<0.5 mmol·L^−1^) following fructose ingestion [75]. It is commonly reported that human skeletal muscle cannot directly oxidise fructose. This is based on human skeletal muscle lacking ketohexokinase (the enzyme responsible for catalysing the phosphorylation of fructose to fructose-1-phosphate). However, in addition to phosphorylating glucose, hexokinase is also able to phosphorylate fructose [76] and when fructose is infused (achieving plasma fructose concentrations of ~5.5 mmol·L^−1^) during exercise, then quantitatively important amounts of fructose (0.3–0.4 g·min^−1^) are likely to be directly oxidised by skeletal muscle [77]. Of course, this is of little relevance to sports nutrition because oral ingestion of fructose rarely results in plasma fructose concentrations exceeding ~0.4 mmol·L^−1^ and thus direct oxidation of fructose is negligible. The reason for a relatively low systemic fructose concentration after fructose ingestion is that fructose is rapidly converted in the intestine and liver to glucose and lactate, which then enter the systemic circulation and delivered to peripheral tissues [78] and/or contribute to liver glycogen synthesis.

When fructose is co-ingested with glucose in large amounts (>0.8 g·min^−1^ each) during exercise, systemic appearance of fructose derived carbohydrate is ~0.5 g·min^−1^ (equally split between fructose-derived glucose and fructose-derived lactate) [78], and the subsequent oxidation of this fructose derived glucose and lactate by skeletal muscle can thus fully account for the higher exogenous carbohydrate oxidation rates seen with glucose–fructose mixtures (sucrose) over glucose alone (Figure 1 and Figure 2). It is unclear what the rate-limiting step in exogenous fructose oxidation is when co-ingested with glucose during exercise, although intestinal absorption is a probable factor. The capacity for humans to absorb dietary fructose is comparatively limited when ingested in isolation. Approximately 60% of individuals display fructose malabsorption after ingestion of large (50 g) fructose loads, with this proportion halved if co-ingested with glucose [79]. Similarly, only 11% of people exhibit fructose malabsorption when ingesting a lower dose of fructose (25 g), with appropriate absorption in almost all cases if that lower dose is ingested with glucose or as sucrose [79]. Therefore, not only does the addition of fructose to glucose ingestion takes advantage of an additional intestinal transport pathway, the ingestion of glucose alongside fructose enhances fructose absorption (via a currently unidentified mechanism) providing a dual mechanism for enhanced carbohydrate delivery. High-fructose diets have been shown to increase intestinal GLUT5 protein content in mice [80]. Therefore, it could be speculated that regularly consuming fructose may enhance the maximal capacity for intestinal fructose absorption, but this remains to be tested in humans.

For athletes, the primary benefit of ingesting glucose–fructose mixtures during exercise is an ability to absorb a greater amount of exogenous carbohydrate to the systemic circulation. This can then be used immediately as a fuel and/or to maintain endogenous carbohydrate stores. More rapid digestion and absorption is also a likely cause of the lower gastrointestinal distress observed with high ingestion rates of isocaloric glucose–fructose mixtures over glucose alone. Lower gastrointestinal distress could, in part, account for some of the performance benefits seen with glucose–fructose co-ingestion [16,81]. The high rates of carbohydrate absorption with glucose–fructose co-ingestion also raise the possibility of enhancing the rate of recovery of endogenous carbohydrate stores post-exercise.

## 5. Glucose–Fructose Co-Ingestion and Recovery from Exercise

### 5.1. Muscle Glycogen Repletion

Glucose and lactate are the primary substrates for muscle glycogen re-synthesis; the latter is able to account for at least 20% of total muscle glycogen re-synthesis following intense exhaustive exercise [82]. Therefore, the availability of carbohydrates (glucose and lactate) to muscle is an important factor in maximising the rate of muscle glycogen repletion and reducing recovery time. Alongside insulinotropic properties, the rate of digestion, intestinal absorption and hepatic metabolism of nutrients are thus important considerations for optimising sports nutrition for rapid post-exercise recovery. Insulin availability is also important for post-exercise glycogen re-synthesis. Insulin increases blood flow to muscle, GLUT4 translocation to the plasma membrane, hexokinase II and glycogen synthase activity [83,84,85,86], all of which contribute to enhanced muscle glucose uptake and glycogen synthesis. A further consideration in the post-exercise period is that elevated catecholamine concentrations may be inhibiting the rise in blood flow and some aspects of insulin signalling in muscle [85,87]. Based on the metabolism of glucose and fructose during exercise (Figure 1 and Figure 2) it could be hypothesised that the greater carbohydrate availability to muscle with ingestion of large amounts of glucose–fructose (sucrose) mixtures could augment post-exercise muscle glycogen repletion rates over isocaloric glucose ingestion alone. In line with this, rates of post-exercise muscle glycogen repletion increase as the rate of carbohydrate ingestion increases, up until ~1 g carbohydrate·kgBM^−1^·h^−1^. This is equivalent to ~1.2 g·min^−1^ for a 72-kg athlete and is therefore in good agreement with the maximal rate of glucose (polymer) digestion and intestinal absorption during exercise (Figure 2). This provides further support for the rationale that carbohydrate delivery to muscle (controlled by digestion, absorption and hepatic metabolism) could be a limiting factor in post-exercise muscle glycogen repletion with carbohydrate feedings.

**Figure 3:**
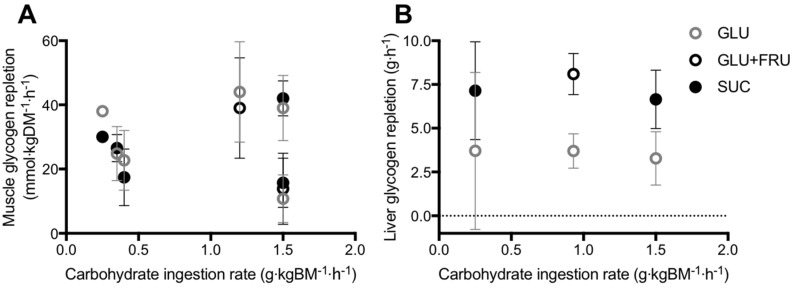
Post-exercise skeletal muscle (**A**) and liver (**B**) glycogen repletion rates in all published studies that have directly compared glucose (polymer) ingestion alone (GLU), vs. either glucose plus fructose co-ingestion (GLU+FRU), or sucrose ingestion (SUC). Bars represent means ± 95% confidence intervals (calculated when sufficient data were available). Data extracted from references [7,85,86,87,88,89,92].

Studies that have directly compared the ingestion glucose–fructose mixtures (or sucrose) vs. glucose (polymers) alone on post-exercise muscle glycogen repletion, have employed carbohydrate ingestion rates ranging from 0.25 to 1.5 g·kgBM^−1^·h^−1^, across two to six hours of recovery [7,88,89,90,91,92]. Across this wide range of carbohydrate ingestion rates, post-exercise ingestion of glucose–fructose (sucrose) mixtures do not appear to accelerate muscle glycogen repletion when compared to glucose (polymer) ingestion alone (Figure 3A). However, lower insulinaemia was reported with glucose–fructose (sucrose) co-ingestion in most [7,88,89,90], but not all [91,92] studies. Therefore, similar muscle glycogen storage appears possible with glucose plus fructose ingestion, compared to glucose ingestion alone, even when insulin availability is lower. It has been suggested that due to the hepatic metabolism of fructose, less glucose may be retained in the liver with glucose–fructose (sucrose) mixtures and more glucose is made available for muscle to be utilised for glycogen re-synthesis, thus offsetting the lower insulin concentrations [89]. 

A further addition to this hypothesis could be that fructose co-ingestion with glucose also provides lactate as an additional fuel source for muscle. Lactate can then be used for muscle glycogen synthesis and/or be oxidised [93], directing more glucose towards muscle glycogen synthesis. Consistent with this, plasma lactate concentrations are higher with glucose–fructose (sucrose) ingestion in post-exercise recovery, when compared to glucose alone, in all [88,90,91,92] but the lowest [7] carbohydrate ingestion rates. This raises the question as to whether providing additional substrate for liver glycogen synthesis (e.g., via galactose co-ingestion) and/or stimulating insulinaemia (e.g., via amino acid co-ingestion) can further accelerate muscle glycogen repletion rates with glucose–fructose mixtures over glucose (polymers) alone. One study has directly compared protein plus sucrose co-ingestion vs. sucrose ingestion alone with high carbohydrate ingestion rates (~1.25 g·kgBM^−1^·h^−1^) and found no difference in muscle glycogen repletion rates. However, arterial glucose concentrations were lower in the protein–sucrose co-ingestion trial [13]. This suggests that either gastric emptying was delayed, and/or splanchnic glucose retention was enhanced with protein co-ingestion. It is therefore currently unknown whether the addition of insulinotropic amino acids [that do not delay gastric emptying [94]] to glucose–fructose (sucrose) mixtures may augment muscle glycogen re-synthesis at high carbohydrate ingestion rates (1.5 g·kgBM^−1^·h^−1^). Combining amino acids with high ingestion rates of glucose–fructose mixtures could take better advantage of high rates of intestinal absorption and the capacity to deliver exogenous carbohydrate to the circulation in combination with higher insulin availability (Figure 2).

Whilst current evidence does not indicate that post-exercise muscle glycogen repletion is accelerated by glucose–fructose co-ingestion over glucose alone, this is achieved with lower gastrointestinal issues. Ingestion of large amounts of carbohydrates is associated with gastrointestinal distress. This could directly reduce the capacity to perform optimally in a subsequent bout of exercise and/or reduce the capacity to tolerate large amounts of carbohydrate ingestion to achieve a muscle glycogen repletion target. The ingestion of isocaloric amounts of glucose–fructose (or sucrose) mixtures, compared to glucose (polymers) alone, reduces ratings of gastrointestinal distress when large amounts of carbohydrate (1.5 g·kgBM^−1^·h^−1^) are ingested over a short-term recovery period (5 h) [90,92].

### 5.2. Liver Glycogen Repletion

In contrast to muscle, the liver is able to synthesize glucose in meaningful quantities from 3-carbon precursors such as glucogenic amino acids, galactose, fructose, glycerol, pyruvate and lactate, in addition to the direct pathway involving intact hexose units [5]. With this in mind, there is potentially a stronger hypothesis for glucose–fructose co-ingestion accelerating liver glycogen repletion over glucose ingestion alone. In addition to higher rates of carbohydrate digestion and absorption, the liver could make use of the ingested fructose for liver glycogen synthesis. Few studies have directly compared glucose plus fructose (sucrose) ingestion with glucose (polymer) ingestion alone, on post-exercise liver glycogen repletion (Figure 3B) [7,90,95]. From these studies, it is apparent that when glucose is ingested alone, the rate of post-exercise liver glycogen repletion is ~3.6 g·h^−1^. Based on the limited number of studies available this does not appear to be dependent on the ingestion rate of glucose (Figure 3B). This may be due to differences in the degree of post-exercise liver glycogen depletion, which appears to be a major driver of liver glycogen synthesis rates [5]. Furthermore, there is large inter-individual variability in basal liver glycogen concentrations [49] and therefore it is recommended that within-subject designs are used to clearly establish the dose-response relationship between post-exercise carbohydrate ingestion and liver glycogen repletion.

When fructose is co-ingested with glucose (either as free glucose plus free fructose, or as sucrose), the rate of liver glycogen repletion is typically ~7.3 g·h^−1^, approximately double the rate seen with glucose ingestion alone (Figure 3B). This effect is clearest when the carbohydrate ingestion rate exceeds 0.9 g·kg body mass^−1^·h^−1^ (Figure 3B). Furthermore, the accelerated liver glycogen repletion rate is consistent when glucose and fructose are either co-ingested as their free monomers, or as the disaccharide sucrose (Figure 3B). The majority of these studies again report lower insulinaemia during post-exercise recovery with glucose–fructose co-ingestion vs. glucose ingestion alone [7,90,95]. It is currently unknown whether the addition of insulinotropic proteins to carbohydrate ingestion can augment post-exercise liver glycogen repletion. It has been speculated that the co-ingestion of protein and fat could also accelerate liver glycogen repletion by increasing gluconeogenic precursor availability [5]. However, on the basis that dietary fat can delay gastric emptying [96], rapidly absorbed amino acids/proteins would be preferable to fat as an option to explore in post-exercise recovery.

Only one study to date has determined post-exercise muscle *and* liver glycogen repletion with the ingestion of large amounts of carbohydrate (>1 g·kgBM^−1^·h^−1^) [90]. Over a five-hour recovery period, ~560 g of carbohydrate was consumed as either glucose (polymers) or sucrose. Based on the maximal rates of digestion, absorption and hepatic release (Figure 2) it could be expected that glucose ingestion would deliver ~360 g to the circulation over the recovery period, compared to ~510 g with sucrose ingestion. In spite of this theoretical 150 g surplus of carbohydrate, only an extra 17 g of glycogen was stored (net) in the liver, and no additional glycogen was stored (net) in muscle (numerical difference of <0.9 g·kg muscle^−1^). It could be speculated that the additional carbohydrate was either oxidised, converted to lipid and/or stored in minor amounts in other glycogen containing tissues such as the kidneys, brain, heart and even adipose tissue [97,98,99]. Fructose plus glucose ingestion accelerates liver glycogen repletion rates over glucose ingestion alone. This acceleration is likely due to the preferential hepatic metabolism of fructose and/or faster digestion and absorption kinetics with glucose plus fructose ingestion, when compared to glucose ingestion.

## 6. Conclusions and Recommendations

The rapid recovery of both muscle and liver glycogen stores after prolonged exercise are important determinants of the capacity to perform a subsequent bout of moderate- to high- intensity exercise. The repletion of liver and muscle glycogen stores is limited by the systemic availability of carbohydrates and glucogenic precursors, along with insulinaemia, the balance of which varies depending on the scenario. The rate of appearance of ingested glucose in the circulation appears to be limited by the capacity of intestinal transporters. Since intestinal fructose absorption utilises a different transport mechanism, combining the ingestion of fructose and glucose takes advantage of both transport mechanisms, thereby increasing the total capacity to absorb carbohydrates. Post-exercise muscle glycogen repletion rates can be maximised by frequent ingestion of carbohydrate throughout recovery at a rate of ≥1.2 g·kg body mass^−1^ every hour, with no further acceleration of glycogen repletion rates if fructose (or sucrose) forms part of the ingested carbohydrate. However, when sufficient carbohydrate is consumed to maximise muscle glycogen replenishment after exercise, the ingestion of glucose plus fructose (sucrose) can minimise gastrointestinal distress. The combined ingestion of glucose plus fructose (or sucrose) during post-exercise recovery strongly enhances liver glycogen repletion rates, but there is currently insufficient evidence to provide guidelines on the carbohydrate ingestion rates required to specifically maximize liver glycogen repletion. When rapid recovery from prolonged exercise is a key objective, and maximal performance is required within 24 h, it is advised to consume more than 1 g carbohydrate^−1^·kg body mass^−1^·h^−1^, starting as soon as possible after exercise and at frequent intervals thereafter (i.e., every 30 min). When ingested in the form of glucose–fructose mixtures (or sucrose), not only is this ingestion rate more tolerable due to lower gut discomfort but total body glycogen status can also be enhanced over glucose (polymer) ingestion alone, due to greater liver glycogen repletion.

## Figures and Tables

**Table 1:** Common dietary carbohydrates, their constituent monomers and major intestinal transport proteins.

Carbohydrate	Chain Length	Constituent Monomers	Bonds	Apical Membrane Intestinal Transport Protein(s)
Glucose	1	-	-	**SGLT1**; GLUT2; GLUT12
Fructose	1	-	-	**GLUT5**; GLUT2; GLUT7; GLUT8; GLUT12
Galactose	1	-	-	**SGLT1**; GLUT2
Maltose	2	Glucose + Glucose	α-1,4-glycosidic	**SGLT1**; GLUT2; GLUT8/12
Sucrose	2	Glucose + Fructose	α-1,2-glycosidic	**SGLT1**; **GLUT5**; GLUT2; GLUT7; GLUT8 GLUT12
Isomaltulose	2	Glucose + Fructose	α-1,6-glycosidic	**SGLT1**; **GLUT5**; GLUT2; GLUT7; GLUT8 GLUT12
Lactose	2	Glucose + Galactose	β-1,4-glycosidic	**SGLT1**; GLUT2; GLUT12
Maltodextrin	~3–9	Glucose + Glucose…	α-1,4-glycosidic	**SGLT1**; GLUT2; GLUT12
Starch	>9 (typically >300)	Glucose + Glucose…	α-1,4- and α-1,6-glycosidic	**SGLT1**; GLUT2; GLUT12

Major transport proteins are highlighted in bold. GLUT, glucose transporter; SGLT, sodium-dependent glucose transporter. Table comprised using information from references [18,19,20,21,22,23].
